# Should a Cardiac Surgeon Blame Himself for Replacing a Mitral Valve?

**DOI:** 10.5935/1678-9741.20160074

**Published:** 2016

**Authors:** Taylan Adademir, Mete Alp

**Affiliations:** 1 Kosuyolu Yuksek Ihtisas Training and Research Hospital, Istanbul, Turkey.

Since the beginning of our residency, we have heard, read and learned a lot about the importance of repair for mitral valve. Besides, we have attended a lot of meetings describing several repair techniques for different pathologies. Both European and American guidelines offer mitral valve repair whenever it is possible and they even do not suggest surgery under some circumstances if the likelihood of successful repair is not more than 95%. Papers from Europe and United States declare up to 70% repair rate for mitral valve and the trend is increasing each year^[1,2]^.

Is it the case for us? As junior cardiac surgeons, we feel sorry for each valve we replace in our centre. That's why we checked our databases to answer the question, "what are we doing to the mitral valve?" The result was far beyond the results of developed countries. We repaired 28% of mitral valves in 2014. This was almost 50% more than the year 2012 as we repaired only 20% of mitral valves but still too low. Low for whom? Developed countries...

In a speech during the 28^th^ European Association for Cardio-Thoracic Surgery Annual Meeting, Maldonado reported the rate of mitral repair in Latin America, and the results were almost the same as ours. Surgeons in Colombia, Chile, Mexico and Brazil repaired 30%, 32%, 39% and 42% of mitral valves, respectively, during 2013. What does this mean? Developing surgeons! Don't they know how to repair mitral valves in developing countries? It shouldn't be the case as each of the above countries has worldwide known and experienced surgeons, for example, the Brazilian Cardiac Surgery Society has more than 1200 surgeons performing around 70 thousand cardiac operations every year.

Etiology of mitral valve disease is the most probable answer for the difference between developing and developed countries. Degenerative mitral valve regurgitation is the most suitable target for repair. Euro Heart Survey revealed that 72% of mitral valve diseases were pure mitral regurgitation and the majority (61%) of etiology was degenerative disease of the valve^[1]^. On the other hand, the Turkish registry of heart valve disease showed that only 30% of pure mitral regurgitation was due to degenerative valve disease and the majority has either ischemic or rheumatic origin. Only 15% of the mitral valves were repaired during the registry^[3]^.

As the majority of the valves referring to surgery are not suitable for repair, surgeons in developing countries, such as Turkey, will be replacing them for a long time. As it is the case, chordal preservation should be considered^[4,5]^. Repairable valve pool is so shallow that a relatively inexperienced surgeon may ignore it during his daily practice and replace a suitable valve. That shouldn't be the case as repair is superior to replacement in many ways. That's why all patients with mitral valve disease should be evaluated by an experienced clinician (echocardiographer) for the possibility of repair. Valve repair requires a unique collection of techniques. A valve with an isolated annular dilatation isolated posterior leaflet prolapse (chordal rupture) or isolated posterior leaflet restriction may be repaired by a relatively inexperienced surgeon but an experienced surgeon is needed for isolated anterior leaflet prolapse, bi-leaflet prolapse or diffuse prolapse of posterior leaflet. More complex lesions of mitral valve like, commissural prolapses, Barlow disease with diffuse involvement, rheumatic disease or calcified leaflet/annulus, should be operated by a reference surgeon in a Heart Valve Centre of Excellence^[6]^.

Physical conditions of a Heart Valve Centre of Excellence may be mimicked in a developing country but here comes another question, "who is a reference surgeon?''. Lack of open access database for surgical results makes this question unanswerable in the developing world.

As a conclusion, we believe in mitral valve repair. We don't have as much suitable valve as developed countries so we are going to replace mitral valve for a long time. When a surgeon faces with a diseased mitral valve he/she should give a chance to repair. But one should be aware that ''not all that he/she can do is, what he/she has to do'', he/she must know what he can do but also he/she must know what he can't do. We should know our limitations and don't be afraid of asking for some help. The patient safety must always come first.
